# Development of Chinese College English Teachers’ Psychological Empowerment Scale: A Validation Study

**DOI:** 10.3389/fpsyg.2022.846081

**Published:** 2022-03-22

**Authors:** Pengfei Lei, Jinfen Xu

**Affiliations:** ^1^School of Foreign Languages, Anhui University of Science and Technology, Huainan, China; ^2^School of Foreign Languages, Huazhong University of Science and Technology, Wuhan, China

**Keywords:** college English teaching, college English teachers’ psychological empowerment, English teachers’ professional development, validation study, development of scale

## Abstract

Relevant research shows teachers’ “psychological power” plays a more essential role in promoting their professional development compared with the powers provided by various external factors, and it is therefore held that exploring college English teachers’ individual psychological power is of great significance. To this end, this study investigated college English teachers’ psychological empowerment (CETPE) via the development and validation of CETPE’s scale. Based on our literature review on psychological empowerment and analysis of teachers professional development’s status quo, we made a working definition of CETPE as a system involving perception of their occupation, sense of competence, experiencing of autonomy, judgment of their impact and understanding of their status. In our study, firstly the literature review and the interview with 17 college English teachers were adopted to conceptualize the dimensions of CETPE, and an exploratory factor analysis of data was conducted from 227 college English teachers and then the subsequent confirmatory factor analysis of data collected from another sample of 1030 generated 15 items belonging to five factors. The findings reveal that CETPE is systematically composed of teachers’ positive perception and experience of work meaningfulness, teaching autonomy, occupational competence, professional impact and social status. This study provides a new path for teachers’ professional development via strengthening their intrinsic driving force and thus helps improve the college English teaching effects.

## Introduction

College English teachers are playing an essential role in developing international professionals on the ground that the globalization and internationalization count much on foreign language, more specifically, English. In this sense, High-quality English teachers would be the guarantee for developing the future professionals with foreign language competence. Since the beginning of the 21st century, great achievements have been made in the studies on college English teachers’ professional development (Borg and Liu, 2013; Huang and Guo, 2019; Lei and Medwell, 2020; Xu, 2020) and the external environment for teacher development has therefore been greatly improved (Wang et al., 2021).

Nonetheless, with the improvement of English level in prep-university students, the marginalization of English teaching in college, and the higher demand of English learning in college students, the bewilderment college English teachers feel in the confidence in competence, perception of work meaning and other aspects in their occupation is on the rise, which obviously pose negative influence on intrinsic motivation in their professional development. How to manipulate those bewilderment is of great importance to provide college English teachers with the psychological power in that relevant studies show that teachers’ “psychological power” plays a greater role in promoting their professional development by providing a strong internal driving force compared with those various external powers endowed by the developmental strategies (Kiliańska, 2012; Sagnak, 2012; Khany and Tazik, 2015; Lee and Nie, 2017; Feiz et al., 2019). Therefore, an in-depth exploration on teachers’ “psychological power,” what we call “psychological empowerment,” may provide the enlightenment and inspiration for promoting college English teachers’ professional development and thus improving foreign language education.

## Literature Review

Psychological empowerment is the psychological paradigm of empowerment research (Gkorezis et al., 2011). Put forward in the 1980s, psychological empowerment has been attracting much attention from the scholars, with explorations conducted mainly on its connotation, causes, effects and so on (Thomas and Velthouse, 1990; Mishra and Spreitzer, 1998). And since 1980s, especially the start of this century, the years witnessed the introduction and boom of psychological empowerment in teacher education (Bin, 2017; Lee and Nie, 2017; Yao et al., 2019).

### Definition of Psychological Empowerment

Psychological empowerment, first proposed by Conger and Kanungo (1988), was defined as the improvement of employees’ expectations of their competence, which was similar to self-efficacy proposed by Bandura (1978). Since Conger and Kanungo (1988), many other scholars have proposed their structure model of psychological empowerment.

Thomas and Velthouse (1990) held that individual’s psychological empowerment could be reflected in his or her evaluation of intrinsic motivation at work. In the interpretive model of “intrinsic task motivation” established by the two scholars, individuals’ cognitive evaluations of work involve “meaning,” “competence,” “self-determination,” and “impact.” It is noteworthy that the “individuals’ internal task motivation” not just depends on the external environment (environmental events and intervention), and the internal factors such as individual behavior (activity, concentration, initiative, resiliency, and flexibility) and interpretive style (attributing, evaluating, and envisioning) also play an essential role in the evaluation tasks.

Spreitzer (1995) proposed a four-dimension model based on Thomas and Velthouse’s (1990) cognitive model of empowerment, including “meaning,” “competency,” “self-determination,” and “impact,” but endowed each dimension with the new connotations. The meta-analysis conducted by Seibert et al. (2011) shows that Spreitzer’s theoretical structure model has higher internal consistency. And the psychological empowerment scale developed by Spreitzer has become most widely used scale in social science research, including teachers’ psychological empowerment.

Psychologist Zimmerman (1995) conducted his community-based research on the empowerment and he defines psychological empowerment as the individual’s sense of control, critical awareness of the environment, and active participation in the environment. However, the community psychological empowerment scale developed by him only includes the internal and behavioral components of the individual, lacking the measurement of the interaction components between the individual and the environment, thus the popularization and application of the scale is limited.

The large-scale study on psychological empowerment emerged relatively late around 2006 in China. In terms of the connotation and structure of psychological empowerment, there are few original studies by Chinese scholars, and most of them tend to adapt the scale by referring to the borrowed mature psychological empowerment scales. The research by Chaoping et al. (2006) is more influential. They designed the employees’ psychological empowerment construct by referring to Spreitzer’s research, and now this version of psychological empowerment scale, with high reliability and validity, is widely used in Chinese cultural context.

Such brief review of psychological empowerment research would provide theoretical reference for the study on college English teachers’ psychological empowerment (CETPE).

### Teachers’ Psychological Empowerment

Since the 1980s, empowerment had been introduced from management to education with the progress of educational reform in the Western countries. People came to realize that teachers should be the active leaders and practitioners rather than the passive objects and followers in the school education reform, and teacher empowerment should play an important part in teacher professional development. However, with the in-depth research on teacher empowerment, scholars found that teachers’ individual psychological motivation had an even greater impact on teaching reform effects compared with external social conditions and environmental factors which gave teachers access to relevant information, human resources and decision-making rights (Sagnak, 2012; Lee and Nie, 2017), teachers’ psychological empowerment was naturally and gradually brought about a widespread attention among the scholars and the educationists. However, the large-scale studies on teacher population have yet remained insufficient (Wang and Liu, 2014; Feiz et al., 2019), and the research on foreign language teachers’ psychological empowerment was even more scarce. For such reason, we sorted the literature review concerning the development and relevant research achievements of teacher psychological empowerment, with the hope of providing references for our study on CETPE.

Though teachers’ psychological empowerment was put forward around 30 years ago, there is yet little consensus on its connotation in the academic literature. After reviewing the literature, it can be found that there are two approaches in exploring the connotation of teachers’ psychological empowerment: self-coining approach and borrowing approach. With the former approach many scholars in the field of education defined teachers’ psychological empowerment inventively in their research on teacher development. Maeroff (1989), the earlier scholar studying teachers’ psychological empowerment, believed that teachers’ psychological empowerment should involve the improvement of teacher status, expansion of knowledge and the opportunity to participate in decision-making. Morris and Nunnery (1993) believed that teachers’ psychological empowerment contained self-efficacy, mastery of professional knowledge and power sharing. According to Dee et al. (2003), teachers’ psychological empowerment was teachers’ internal motivation to improve their personal power, and it was essentially a subjective mental state which enabled teachers to control their teaching performance through self-efficacy. In China, psychological empowerment was believed to have an essential impact on teachers’ confidences in their competence and behavior; teachers with high psychological empowerment would fully understand the social management system and actively participate in various activities; and they would more actively realize self-value in the classroom, school and the community (Cao and Lu, 2007). However, the self-coining approach method brought with itself the shortcoming: those definitions are made based on their own understandings and research under different theoretical frameworks from different disciplines, therefore it was difficult to come to a consensus, bringing about the inconvenience to promote the research conclusions. For this reason, our study would extract the common points from these self-coining theories to develop the CETPE scale.

Borrowing approach was to explore the connotation of teachers’ psychological empowerment by referring to the mature psychological empowerment theories in organizational management, sociology and other related fields. For example, Spreitzer’s (1995) four-dimensional model and Zimmerman’s (1995) community-based model are popularly used for reference in the study on teachers’ psychological empowerment. Chinese scholar Wang (2015), based on Spreitzer’s study, systematically discussed the psychological empowerment of teachers in Chinese universities, and she conducted an empirical study to verify the college teachers’ psychological empowerment as meaningfulness, self-efficacy, autonomy, and impact. And the research performed by Wang and Zhang (2011) on Chinese primary and secondary school teachers’ psychological empowerment was mainly based on Zimmerman’s theory, they concluded that teachers’ psychological empowerment is a system of experience (including self-efficacy, impact, status, and autonomy), skills (including skills of decision-making and communication), and behavior (including behaviors that influence teaching and decision-making).

In our study, the CETPE’s connotation will be explored with mixing these two methods, that is, via referring to those mature theories and via analyzing the interview data from college English teachers. The reasons for our research design are as follows: Firstly, to be more reliable. These adopted theories are derived from the other social sciences such as organizational management and behavior science, where the empowerment theories are rather mature and the structure and related scales have higher reliability after being tested by so many researchers; moreover, their procedures of developing the theories are also enlightening for our research. Secondly, to be more valid. Almost any theory has its preconditions and specific contexts in use, in order to figure out the CETPE scale of high validity characterized with Chinese social and cultural context, it is necessary that the in-depth interviews with college English teachers be conducted, the large-scale data be collected and conducted with content analysis, which would ensure the localization and adaptation of the CETPE scale to be developed.

Our study adopted the method combining qualitative research and quantitative research to explore CETPE scale. Specifically, the study would be converged onto the following research questions:

What is scale of psychological empowerment for college English teachers?

## Materials and Methods

**FIGURE 1:**
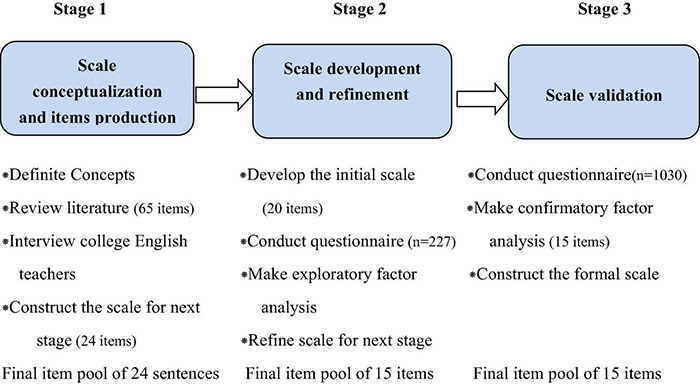
The scale development procedures in our study.

This study would take developing the CETPE scale as its top priority, a rational approach to scale development proposed by Clark and Watson (1995) would be taken in our study, namely, identification of salient concepts or dimensions by referring to the literature and investigating, development of the sets of items for new instrument, and validating the instrument through various forms of testing. Accordingly, our study involves the following procedures: Firstly, to identify the concepts and items of the CETPE scale by referring to the literature review and by interviewing and consulting the 17 college English teachers (Stage 1). Next, scale refinement was conducted based on the data collected from the questionnaire done with 227 college English teachers (Stage 2). The scale was then validated in the study with a sample of 1030 college English teachers from the colleges and universities in China (Stage 3). These studies was elaborated in the following section. Figure 1 summarizes the scale development procedures.

### Procedures and Results

#### Stage 1: Scale Conceptualization and Items Production

##### Defining Concepts

This study, referring to the research achievement of teachers’ psychological empowerment in literature, taking into consideration the social and cultural environment where Chinese college English teachers work, made a working definition of college English teachers’ psychological empowerment (CETPE) as “a cognitive state in which college English teachers believe they have strong power and motivation, which is manifested in teachers’ positive perception and experience of the meaningfulness, self-competence, autonomy, impact and status of the work they are engaged in; it is influenced comprehensively by individuals’ internal factors and external environment factors, and would provide internal driving force for teachers’ professional development and produce positive results.” In this definition, meaningfulness, self-competence, autonomy, impact and status are the five key concepts in CETPE.

##### Producing Items by Reviewing Literature

Using this working definition as the guideline, we took a multi-source approach to generate items interpreting the five concepts’ connotations, with the excluded information as the sixth category “Other” if possible. First, we came back to the related literature that provides the explanation to the concepts to figure out the core information concerning psychological empowerment; Second, we turned to the existing scales proposed by the scholars at home and abroad to get the enlightenment and inspiration, such as Spreitzer’s (1995) Psychological Empowerment Scale, Short and Rinehart’s (1992) Teacher Empowerment Structure Scale (SPES), Wang and Zhang’s (2011) scale and Wang’s (2015) scale and so on. In this way, 62 items were collected which were rather closely related to the five concepts, 13 (meaningfulness), 14 (self-competence), 15 (autonomy), 13 (impact), and 7 (social status), no information belonging to “Other” category.

##### Producing Items by Interviewing Teachers

Then a semi-structure interview was done to the college English teachers in order to check the items we had collected and to complement what did not include in our collection; moreover, the interview was also expected to provide the explanation to the CETPE’s connotations. 17 college English teachers were selected as the voluntary interviewees by taking into consideration the representativeness of the sample, with 5 male teachers and 12 female teachers and their teaching time ranging from 1 year to 32 years, with 2 teaching assistants, 11 lecturers, 3 associate professors and 1 professor; Among them, 3 teachers are doctors, 13 masters and 1 bachelor; and 7 teachers came from universities of science and engineering, 5 from comprehensive universities, 2 from normal colleges, 2 from financial colleges and 1 from medical colleges, which substantially reflected the sex ratio, professional titles and the proportion of different types of universities in Chinese context.

The researcher didn’t determine the specific questions raised in the interview in advance, but only predetermined the interview theme and topic area. In this way, the researcher would not be bound by the existing research results, and would suggest or prompt the interviewed teachers to provide more extensive and in-depth information in the interview process (Dörnyei, 2007). And the researcher conducted interviews by following the “hourglass-type” questioning mode, that is, starting from relatively broad and open questions, gradually narrowing down and deepening into the specific questions, and finally asking for the viewpoints on the specific questions, that is, the “generative” interview method of exhaustively inquiring. One 60-min interview with each teacher, respectively, was conducted; and a second interview of about 30 min with 4 of the subjects was conducted to clarify the content demanding further their confirmation after carefully checking the recordings.

The interview data was collected from October 4th, 2019 to October 16th, 2019, lasting about 2 weeks, 1050 min audio data were collected (after removing irrelevant information to the study), and was transcribed into a text of 17,747 Chinese characters.

Content analysis was employed to process and analyze the interview data. Before the analysis, the researchers were trained until they could design the coding system framework according to psychological empowerment theory and text features; then the researchers were trained to compact the collected qualitative research data into a limited number of concise and comprehensive specific expressions (Krippendorff, 2014); finally, those expressions were modified and refined as the criteria to code all the collected data.

In order to ensure the reliability of the content analysis, two coders (the researcher and a Doctor of Education sharing the same research interest) in this study processed the data by using percentage agreed coefficient, the formula was: pA_0_ = A/n, where the “pA_0_” was the agreed coefficient, “A” was the times for which the two coders agreed on a particular category, “n” was the total number of data units to be analyzed by the two coders in this category, namely, 17 in our study. Take the category of “I feel that I have the skills required for college English teaching” for example, the times of two coders reaching agreement among the sample data of 17 interviewees was 15 (it was counted as one time for repeated occurrence of the same category in the same sample), the coding agreed coefficient of this category was 15/17, namely, 0.882. Table 1 shows the example.

**TABLE 1:** Example of percentage agreed coefficient.

Items	Content categories	Percent agreed (*n* = 17)	Coders	No. 1	No. 2	No. 3	No. 4	No. 5	No. 6	No. 7	No. 8	No. 9	No. 10	No. 11	No. 12	No. 13	No. 14	No. 15	No. 16	No. 17
1	I feel I have the skills required for college English teaching	0.882	Coder 1	1	1	0	1	0	1	1	0	1	1	0	1	1	1	1	0	1
			Coder 2	0	1	0	1	0	1	1	1	1	1	0	1	1	1	1	0	1
2	I am confident that I can help students improve their foreign language skills	0.765	Coder 1	1	0	1	1	1	0	1	1	1	1	1	1	1	1	0	1	0
			Coder 2	1	0	0	1	1	1	1	0	1	0	1	1	1	1	0	1	0
:	:	:	Coder 1	:	:	:	:	:	:	:	:	:	:	:	:	:	:	:	:	:
:	:	:	Coder 2	:	:	:	:	:	:	:	:	:	:	:	:	:	:	:	:	:

*In the coding, “1” means there exist the content in this text; “0” means there does not exist the content in this text.*

In our study, the average agreed coefficient was around 0.8, being high enough to show the reliability of coding met the requirement.

##### Constructing Final Item Pool

After carefully analyzing the interview data, the researcher found that the interviewed teachers held that college English teachers’ perception of their status was also a significant source of their confidence and psychological superiority when communicating with their colleagues and students. Therefore, the connotation of “status” mentioned in this research was different from that in Short and Rinehart’s (1992) study. It mainly referred to the psychological strength teachers sensed when they were appreciated, respected and supported by colleagues and students due to their moral character, dedication and excellent performance.

On the basis of results from our interview and with reference to items from the literature, this study figure out 24 key concepts for the initial CETPE scale by integrating, refining those points. We transferred those key concepts into short sentences, as shown in Table 2.

**TABLE 2:** Descriptions in the initial CETPE scale (translated from the Chinese version).

Items	Key concepts transferred into sentences	Fre.	Percentage of teachers held in total	Percentage agreed coefficient
1	I feel I have the skills required for college English teaching	11	64.7%	0.882
2	I am confident that I can help students improve their foreign language skills	10	58.8%	0.765
3	I feel that my classroom teaching ability is strong	12	70.6%	0.882
4	I don’t feel confident in my academic research	9	52.9%	0.824
5	I think being an English teacher can demonstrate my self-worth	4	23.5%	0.882
6	I think college English is more important to the national development	4	23.5%	0.941
7	I think what I do in teaching is personally meaningful	5	29.4%	0.765
8	I feel responsible for the improvement of students’ foreign language ability	9	52.9%	0.824
9	I perceive the development of my professional skills	5	29.4%	0.882
10	I am aware that national policies provide opportunities for my development	3	17.6%	0.765
11	I feel that my development is valued by the school	3	17.6%	0.824
12	I think the development of my research ability is not ideal	11	64.7%	0.941
13	I hold that the content and methods in English teaching can be chosen by myself	7	41.2%	0.824
14	I feel free to choose how I want my work done	8	47.1%	0.882
15	I think I am able to participate in the policy making in our school	4	23.5%	0.765
16	I believe I can choose the interest and content of the research for my own	3	17.6%	0.882
17	I feel various limitations in my teaching work	10	58.8%	0.824
18	I perceive myself as having a positive impact on the affairs in our school	3	17.6%	0.706
19	I feel that I am respected by other teachers in my school	7	41.2%	0.882
20	I feel respected by students as a college English teacher	13	76.5%	0.941
21	I feel I have the academic influence to some extent	2	11.8%	0.765
22	I feel appreciated by my colleagues for having a positive outlook on life, values and the world	8	47.1%	0.824
23	I feel appreciated by my colleagues for my contributions to English teaching	10	58.8%	0.882
24	I think I can set an example for others as a college English teacher	11	64.7%	0.882

*The total number is 17 in that there were 17 teacher interviewed.*

#### Stage 2: Scale Development and Refinement

In this stage, the initial scale was to be developed based on the CETPE’s description collected from the interview with the college English teachers, and then it was refined by the exploratory factor analysis of the data collected from the questionnaire.

##### Developing the Initial Scale

With many rounds of discussions and modifications with our experts team of 2 professors with doctor degree in psychology, 2 professors with doctor degree in education and 1 professor with doctor degree in social statistics, the agreement was drawn that Items 3, 4, and 24 shared the core information with Items 13, 12, and 22, respectively, and should be integrated; and Item 9 was rather abstract and obscure and could be interpreted with more specific expression in Items 1 or 2. In this way, the 24 CETPE’s descriptions were re-integrated and transferred with more refinement in their diction into a 5-point Likert scale of 20 items (5 = strongly agreed or very satisfied; 1 = strongly disagreed or very dissatisfied) in order to do the questionnaire, as shown in the Table 3 (the original version was in Chinese so that there would be little misunderstanding to interviewed teachers). This initial scale was to be used to collect data to do the exploratory factor analysis for the purpose of refining the scale.

**TABLE 3:** The integrated initial CETPE scale (translated from the Chinese version).

Items
VarA1. I think teaching English in college is meaningful national development
VarA2. I feel I have some influence on making policies or measures in our school
VarA3. I perceive the appreciation from the colleagues in the work
VarA4. I am confident of my ability to be a competent college English teacher
VarA5. I think I have much self-determination in my teaching work
VarA6. I think being college English teacher is a noble profession
VarA7. I perceive the positive impact on my students
VarA8. I am aware that I am taken as the professionals at English teaching in the school
VarA9. I am upset I am not equipped with the ability to deal with the challenges from the work (-)
VarA10. I think I can make my own decision to attend academic conferences or training
VarA11. I feel teaching English in college is very important to me
VarA12. My opinion tends to be taken seriously in solving the problems
VarA13. I perceive the support and respect from colleagues in the work
VarA14. I think I have the freedom to get the research work done the way I choose
VarA15. I believe I have possessed the knowledge and skill needed in college English teaching
VarA16. I think teaching English in college does not reflect my personal value (-)
VarA17. I feel I have the positive impact on the colleagues around me
VarA18. I perceive the respect from the students
VarA19. I think I am able to work with the guidance of scientific research
VarA20. I perceive various restrictions and lack of freedom in my professional training (-)

*(-) means reverse scores.*

##### Conducting the Survey

A total of 263 samples were collected from the in-service college English teachers from 15 colleges and universities of different types and levels nationwide by simple random sampling in that the sample size were not large and the chance with which every teacher would be selected was the same. All the teachers answered the questionnaire voluntarily and anonymously and received a letter of thanks after the questionnaire completion in paper form or on line by the APP. In order to ensure the data quality, this study designed the screening criteria by referring to the effective questionnaire screening criteria commonly used in the academic research, namely, those questionnaires with (1) more than 10% missing items, (2) more than 1/3 items selecting “basically agree,” (3) inconsistent answers to the reverse questions, (4) answering time less than 240 s set by the researchers in our pre-tests (Revilla et al., 2016), would be regarded as invalid and deleted. According to these criteria, 227 valid samples were eventfully obtained, with detailed information of the participants shown in Table 4.

**TABLE 4:** Background information of the teacher participants (*n* = 227).

Variables	Content	Fre.	Percent (%)
Sex	Male	53	23.58
	Female	174	76.42
Age	Under the age of 30	14	6.11
	Between 31 and 40	128	56.33
	Between 41 and 50	63	27.95
	Above the age of 50	22	9.61
Teaching time	Less than 5 years	22	10.04
	Between 6 and 10 years	40	17.47
	Between 11 and 15 years	74	32.75
	More than 15 years	91	39.74
Educational background	Bachelor	22	10.04
	Master	180	79.04
	Doctor	24	10.48
	Other cases	1	0.44
Professional titles	Teaching assistants	10	4.37
	Lectures	118	51.97
	Associate professors	89	39.3
	Professors	10	4.37
Types of universities	Universities of science, technology, agriculture, and medicine	70	31
	Universities of liberal arts	20	8.73
	Normal university	21	9.17
	Universities of arts and physics	2	0.87
	Comprehensive universities	87	38.43
	Other cases	27	11.79

##### Refining the Scale

In this study, SPSS 19.0 software was used to make item analysis, reliability and validity test on the initial scale.

First, the item analysis was done to remove the items which did not meet the statistical standard. According to the item analysis results, among the 20 items in the initial CETPE scale, items VarA9 (*t* = −0.111, *P* = 0.912) and VarA16 (*t* = −1.608, *P* = 0.110) had lower *t*-values by using the extreme grouping method and did not reach the significance level (*P* < 0.05); in addition, their correlation with the total score of all variables were only −0.072 and −0.139, respectively, failing to reach the level of significance (*P* < 0.05). Therefore, the results indicated that for VarA9 and VarA16, the differentiation degrees were not high enough and should be deleted. The remaining 18 items demonstrated good differentiation.

Next, the reliability analysis was done to the scale. The overall Cronbach’s α coefficient of the scale was 0.846, higher than 0.8, as shown in Table 5. The result indicated that the internal consistency of the scale was quite ideal. Without removing any measurement item, the total correlations of individual items were all higher than 0.4; meanwhile, deleting any item did not lead to the increase of Cronbach’s α coefficient, therefore, it was indicated that the scale’s internal consistency reliability was at a rather high level, meeting the requirements of subsequent empirical analysis (Hair et al., 2018).

**TABLE 5:** Reliability analysis of the initial CETPE Scale (*N* = 227).

Variables	Corrected item-total correlation	Cronbach’s alpha if item deleted	Cronbach’s alpha
VarA1	0.506	0.836	0.846
VarA2	0.544	0.844	
VarA3	0.594	0.833	
VarA4	0.435	0.839	
VarA5	0.486	0.837	
VarA6	0.522	0.835	
VarA7	0.523	0.836	
VarA8	0.523	0.835	
VarA10	0.406	0.841	
VarA11	0.610	0.831	
VarA12	0.469	0.838	
VarA13	0.620	0.832	
VarA14	0.580	0.832	
VarA15	0.483	0.837	
VarA17	0.535	0.834	
VarA18	0.614	0.831	
VarA19	0.460	0.838	
VarA20	0.589	0.880	

In KMO and Bartlett’s test, the KMO value was 0.880, higher than the minimum value 0.6, and the Bartlett sphere test also reached the significance level (0.000, *P* < 0.05), indicating that this data was suitable for factor analysis.

In the exploratory factor analysis, five common factors with eigenvalue greater than 1 were obtained after two exploratory factor analyses according to item deletion criteria, and the cumulative explicable variance was 67.352%. In this process, three items were deleted, namely VarA8, VarA10, and VarA18. After the revision, the CETPE scale’s reliability and validity met the statistical requirements. It was noteworthy that VarA20, carrying a negative factor loading (−0.803), was included in Autonomy because it was designed as the reverse statement to the connotation of “Autonomy.” With reference to the literature review, the interview and content analysis we have conducted in Stage 1, we named these five factors as Status, Competence, Impact, Autonomy, and Meaningfulness, as shown in Table 6.

**TABLE 6:** Exploratory factor analysis of the revised CETPE scale (*N* = 227).

Variables	The extracted common factor variance	Factors				
		F1 status	F2 competence	F3 impact	F4 autonomy	F5 meaningfulness
VarA17	0.684	0.732				
VarA3	0.669	0.719				
VarA13	0.665	0.530				
VarA15	0.673		0.765			
VarA19	0.631		0.758			
VarA4	0.584		0.645			
VarA12	0.764			0.819		
VarA2	0.679			0.814		
VarA7	0.667			0.650		
VarA20	0.668				−0.803	
VarA5	0.704				0.769	
VarA14	0.687				0.698	
VarA11	0.716					0.751
VarA1	0.701					0.716
VarA6	0.648					0.582
Eigenvalue		5.619	1.916	1.347	1.124	1.007
Variance contribution (%)		35.116	11.977	8.419	7.027	4.812
Cumulative variance contribution (%)		35.116	47.093	55.513	62.540	67.352

*Extraction method: principal component.*

*Rotation method: Orthogonal rotation method with Kaiser standardization.*

*Rotation converges after 7 iterations.*

*The factor load without numbers is less than 0.5.*

In this way, the study proposed the hypothesized model of CETPE that there were five latent variables. The relationship among latent variables and the observed variables (the measurement model) would be verified using structural equation modeling (SEM).

#### Stage 3: Scale Validation

In this stage, the scale would be validated using SEM.

##### Conducting the Questionnaire

In order to validate the scale, another group of college English teachers were taken as the research sample. We determined the sample size to be about 1500.

The 15 items in the CETPE Scale obtained in Stage 2 were re-entitled, and the multiple choices (5 = strongly agreed or very satisfied; 1 = strongly disagreed or very dissatisfied) were added to each of the items to form the formal 5-point Likert scale, as shown in Table 7.

**TABLE 7:** The formal CETPE scale (translated from the Chinese version).

Items
CETPE 1. I think teaching English in college is meaningful for national development
CETPE 2. I feel I have some influence on making policies or measures in our school
CETPE 3. I perceive the appreciation from the colleagues in the work
CETPE 4. I am confident of my ability to be a competent college English teacher
CETPE 5. I think I have much self-determination in my teaching work
CETPE 6. I think college English teacher is a noble profession
CETPE 7. I perceive the positive impact I have on my students
CETPE 8. I feel teaching English in college is very important to me
CETPE 9. My opinion tends to be taken seriously in solving the problems
CETPE 10. I perceive the support and respect from colleagues in the work
CETPE 11. I think I have the freedom to get the research work done the way I choose
CETPE 12. I believe I have possessed the knowledge and skill needed in college English teaching
CETPE 13. I feel I have the positive impact on the colleagues around me
CETPE 14. I think I am able to work with the guidance of scientific research
CETPE 15. I perceive various restrictions and lack of freedom in my professional training (-)

*(-) means reverse scores.*

The researcher conducted a formal questionnaire from October 2019 to January 2020, involving the colleges and universities in 26 provinces, municipalities and autonomous regions of China. Similarly, all the teachers answered the questionnaire voluntarily and anonymously and received a letter of thanks for their participation. Cluster sampling was adopted because the sample size was large, moreover, the features of the group of college English teachers summarized from the sampling in Stage 2 provided useful reference to cluster sampling. A total of 1426 questionnaires were collected; After strictly screening of these questionnaires according to the valid questionnaire standards developed in this study, 1030 valid questionnaires were finally obtained, the detailed information of the participants was shown in Table 8.

**TABLE 8:** Background information of the teachers participants (*n* = 1030).

Variables	Content	Fre.	Percent (%)
Sex	Male	148	14.4
	Female	882	85.6
Age	Under the age of 30	66	6.4
	Between 31 and 40	555	53.9
	Between 41 and 50	328	31.8
	Above the age of 50	81	7.9
Teaching time	Less than 5 years	116	11.3
	Between 6 and 10 years	174	16.9
	Between 11 and 15 years	339	32.9
	More than 15 years	401	38.9
Educational background	Bachelor	75	7.3
	Master	837	81.3
	Doctor	118	11.5
Professional titles	Teaching assistants	68	6.6
	Lectures	625	60.7
	Associate professors	284	27.6
	Professors	50	4.9
	Other cases	3	0.3
Types of universities	Universities of science, technology, agriculture, and medicine	300	29.1
	Universities of liberal arts	74	7.2
	Normal university	184	17.9
	Universities of arts and physics	23	2.2
	Comprehensive universities	438	42.5
	Other cases	11	1.1

After the questionnaire, in order to obtain deeper interpretation of the collected data, a total of 22 teachers were selected to do the structured interviews on telephone; the transcript of the recording with their authorization was used as the important supplement and verification for data analysis.

##### Processing the Data

SPSS 19.0 and Amos 24.0 software were used for data processing. Generally, it is the premise for subsequent data processing and analysis that the sample data should demonstrate state distribution. In this study, the distribution analysis in SPSS frequency analysis was applied to calculate the skewness and peak value of each item, all of which were within (−1, 1), indicating that the samples met the basic conditions for subsequent data processing. In this part, many important estimates concerning the quality of default model were displayed.

The standardized regression weights, also called factor loading, represents the influence of common factors on the observed variables. The estimates of factor loading calculated from our collected data were shown in Table 9.

**TABLE 9:** Standardized regression weights: (group number 1 – default model).

			Estimate				Estimate
CETPE1	<−−−	Me	0.683	CETPE2	<−−−	Im	0.700
CETPE6	<−−−	Me	0.736	CETPE7	<−−−	Im	0.685
CETPE8	<−−−	Me	0.780	CETPE9	<−−−	Im	0.889
CETPE4	<−−−	Co	0.663	CETPE3	<−−−	St	0.651
CETPE12	<−−−	Co	0.651	CETPE10	<−−−	St	0.703
CETPE14	<−−−	Co	0.734	CETPE13	<−−−	St	0.720
CETPE5	<−−−	Au	0.847				
CETPE11	<−−−	Au	0.822				
CETPE15	<−−−	Au	0.619				

As can be seen in Table 9, most of the factor loading are between 0.50 and 0.95, indicating that the fit of the model with the data is rather good.

Covariances and correlations in the default model demonstrate the covariant relationship among the latent variables.

As shown in Table 10, the estimates of covariances were not 0 at the significant level, indicating there was significant covariant relationship among the latent variables; meanwhile, the correlations coefficients among the five variables were all higher than 0.4 at the significant level, meaning that there probably would be a higher-order common factor.

**TABLE 10:** Covariances and correlations (group number 1 – default model).

			Estimate	C.R.	*P*					Estimate
Me	<−−>	Co	0.145	9.205	***	par_11	Me	<−−>	Co	0.553
Me	<−−>	Au	0.312	11.523	***	par_12	Me	<−−>	Au	0.553
Me	<−−>	Im	0.247	11.122	***	par_13	Me	<−−>	Im	0.545
Co	<−−>	Au	0.143	8.679	***	par_14	Co	<−−>	Au	0.429
Co	<−−>	Im	0.118	8.716	***	par_15	Co	<−−>	Im	0.44
St	<−−>	Co	0.146	10.259	***	par_16	St	<−−>	Co	0.719
Au	<−−>	Im	0.345	11.871	***	par_17	Au	<−−>	Im	0.6
St	<−−>	Au	0.295	12.86	***	par_18	St	<−−>	Au	0.677
St	<−−>	Im	0.261	12.116	***	par_19	St	<−−>	Im	0.745
St	<−−>	Me	0.230	11.587	***	par_20	St	<−−>	Me	0.667

*** Means “reach a significant level 0.05”.

Standardized residual covariances are the index to show the model’s goodness of fit with the collected data. The cut-off points of Standardized Residual Covariances are mostly regarded as not higher than 2.58 in their absolute values (Byrne, 2001). Table 11 is the result of standardized residual covariances in our study.

**TABLE 11:** Standardized residual covariances (group number 1 – default model).

	CETPE13	CETPE10	CETPE3	CETPE9	CETPE7	CETPE2	CETPE15	CETPE11	CETPE5	CETPE14	CETPE12	CETPE4	CETPE8	CETPE6	CETPE1
CETPE13	0														
CETPE10	–0.103	0													
CETPE3	0.075	–0.008	–0.005												
CETPE9	1.039	–0.98	–0.737	0											
CETPE7	–0.494	0.025	1.054	–0.172	0.37										
CETPE2	1.08	–1.398	0.172	0	–0.158	0									
CETPE15	–2.408	–1.635	–0.928	–0.517	–0.898	0.43	0								
CETPE11	1.153	0.988	–0.474	1.277	–1.744	0.555	0.791	0							
CETPE5	0.032	0.396	0.591	1.711	–1.489	1.887	0.171	–0.306	0						
CETPE14	–0.102	0.548	–0.343	–1.72	0.539	–1.363	–1.408	0.186	0.793	0					
CETPE12	0.241	–0.432	0.371	–0.636	1.012	–1.067	–0.099	0.595	0.518	0.507	0				
CETPE4	–0.612	–0.094	0.328	–1.078	1.474	–1.497	–0.635	–0.972	0.963	0.572	0.844	0.868			
CETPE8	–1.249	0.179	–0.736	–0.852	0.522	–1.552	–0.515	–0.869	–0.347	–1.163	–1.444	0.266	0		
CETPE6	–0.683	0.966	–0.333	–0.557	0.285	–1.108	0.432	–0.129	0.994	–0.185	–0.773	0.105	0.574	0.36	
CETPE1	–1.691	1.784	1.699	–1.52	0.814	–0.224	1.054	–0.998	0.515	–0.278	–0.222	0.647	0.431	–0.508	0

It can be seen in Table 11 that no values in the data are greater than 2.58, indicating that the model has higher internal quality.

To test the CETPE scale’s reliability, Jamovi 0.7 was used in our study. The Cronbach’s α coefficient and the McDonald’s ω coefficient of the scale were obtained, 0.835 and 0.839, respectively, which indicated that the reliability reached the statistical standards (Viladrich et al., 2017).

##### Validating the Scale

To verify the scale’s structure validity is actually to evaluate goodness-of-fit indices between the formal questionnaire data and the hypothetical model which was set in Stage 2. Amos conducted tests based on chi-square statistics. However, chi-square statistics is sensitive to sample size, so other supplementary fitting indices would be taken as the references. The valid sample size of this study was rather large, therefore, the index of Chi-square degree of freedom ratio would be appropriately lowered when judging the model’s goodness-of- fit. We presented the model fit summary obtained in the confirmatory factor analysis.

The researcher analyzed the measurement invariance with SEM, the comparison was made between the male participants and female participants in our questionnaire, and it was found that *p* value of measurement residuals in nested model comparisons was >0.05, indicating that there was a satisfactory measurement invariance in the internal structure of our questionnaire.

The quality of the scale model was tested with convergent validity, discriminant validity and criterion validity. As to convergent validity, in our study, the factor loading of all the variables were between 0.619 and 0.889, higher than 0.5, and *t* value higher than 1.96; the five latent variables’ AVE is between 0.548 and 0.654, greater than 0.50, and the CR between 0.919 and 0.967, great than 0.7. The results indicated the convergent validity of the scale is rather good. As to discriminant validity, in our study, with Pearson Correlation Analysis and the square root of AVE, it showed that Factor 1’s square root of AVE was 0.813, greater than the relation coefficients between Factor 1 and the other 4 factors (with 0.767 the highest); Factor 2’s was 0.84, greater than the relation coefficients between Factor 2 and the other 4 factors (with 0.753 the highest); similarly, Factor 3’s, 0.907; Factor 4’s, 0.921; Factor 5’s, 0.864, greater than they were with other relation coefficients. The result indicated the good discriminant validity of the model. Moreover, we developed the dimensions and items of the scale by referring to the authoritative theories in psychological empowerment research, such as Spreitzer’s and Zimmerman’s, to ensure the criterion validity of the model.

Generally, the lower the value of RMSEA, the better the model would be, more specifically, with the value less than 0.05 being the best fit, more than 0.1 being the bad fit.

In Table 12, RMSEA was 0.068 in our study, indicated that the default model of the scale had a good fit with the observed data according to the cut-off points.

**TABLE 12:** RMSEA.

Model	RMSEA	LO 90	HI 90	PCLOSE
Default model	0.068	0.062	0.074	0.000
Independence model	0.238	0.233	0.243	0.000

Akaike information criterion (AIC) is mainly taken to judge whether the number of parameters estimated by the theoretically hypothetical model is concise enough.

In Table 13, the AIC value (270.00) of the default model was lower than that of the Saturated model (548.897) and the Independence model (6294.597), indicating that the default model was acceptable.

**TABLE 13:** AIC.

Model	AIC	BCC	BIC	CAIC
Default model	270.000	274.265		
Saturated model	548.897	550.824		
Independence model	6294.597	6295.545		

More indices that indicate the goodness of fit of model are shown in Table 14 together with their cut-off points, and the values of those indices obtained in our study can be found in this table.

**TABLE 14:** Fitting indices of CETPE scale’s SEM.

	x^2^/df	RMSEA	RFI	NFI	TLI	IFI	CFI
Overall model fit standards	<2, good 3 < x^2^/df < 5, acceptable	<0.05, good; <0.1 acceptable	>0.90	>0.90	>0.90	>0.90	>0.90
First order five dimensional model	4.765	0.068	0.903	0.932	0.918	0.943	0.942

It could be seen from Table 14 that, most fitting indices of the first-order five-dimension model were within the acceptable range and relatively ideal in comparison with those in the alternative model, and the first-order five-dimension model had a satisfactory goodness-of-fit with the hypothetical model.

**FIGURE 2:**
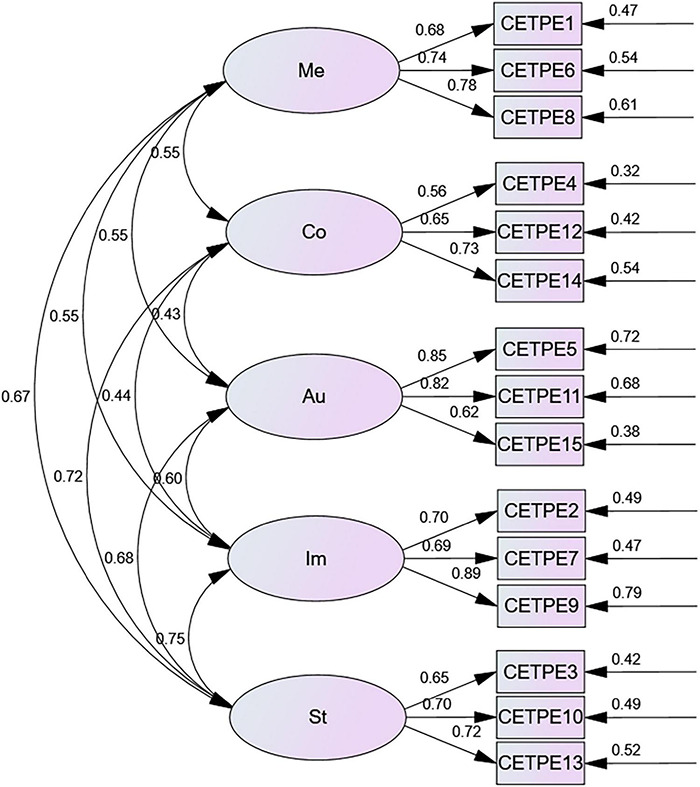
CETPE’s five dimensional model. Among the variables of the model, Me, Meaningfulness; Co, Competence; Au, Autonomy; Im, Impact; St, Status; CETPE, college English teachers’ psychological empowerment.

The standardized estimated model after fitting was shown in Figure 2. As is shown in the figure, CETPE is composed of five components, namely, Meaningfulness, Competence, Autonomy, Impact and Status, which are interrelated to each other to form a system with the coefficients showing the connections (more than 0.4); for each component (latent variable), there were three items (observed variables) to indicate the connotations, respectively.

## Discussion and Conclusion

The primary objective of this study was to develop the scale for CETPE. Utilizing an empirical validation design, it was expected that the CETPE’s connotations could be more localized and contextualized (Wang et al., 2013), and that for the components of the scale, there would be appropriate interpretations. According to the analysis of the literature review, the data from interviews and questionnaires conducted in our research, it can be safely demonstrated that CETPE mainly contains teachers’ perceptions of meaningfulness, competency, autonomy, impact and status in college English teaching and the five dimensions were endowed with specific embodiment in college English teachers’ professional development environment.

Meaningfulness is expressed by the judgments made by college English teachers on the value of what they are doing based on the standards they hold (Spreitzer, 1995). Meaningfulness reflects the matching of teachers’ personal values, beliefs and practical work behaviors with their roles as teachers. College English teachers who consider their work as meaningful would actively dedicate themselves to teaching career (0.74) and national development (0.68), and consciously strive to realize their personal value as college English teachers in the new era (0.78). Teachers who perceive their job meaningfulness would seldom suffer from burnout or turnover intention (Ding, 2021).

Competence mainly refers to teachers’ confidence in whether they are able to fulfill the duties and perform the tasks with their professional knowledge, skills and quality. For college English teachers, they should not only be psychologically empowered with their mastery of the content knowledge, pedagogical knowledge, pedagogical content knowledge, student knowledge, educational environment knowledge and educational purpose knowledge (0.56), but also be confident of their basic language teaching skills (0.65) and scientific research quality (0.73). The new ecology of college education poses various challenges and much higher requirements for college English teachers’ competence. Only when the college English teachers have the positive attitude and experience toward demonstrating and evaluating their competence at various facets, can they remain committed to and motivated in their professional development. As is shown in the study conducted by Baleghizadeh and Goldouz (2016) and Zhu et al. (2019), the teachers’ innovative performance in teaching and researching as their professional learning communities could be improved by increasing teachers’ psychological empowerment of competence.

Autonomy which has been handled with as an ability to act freely (Orakcı and Gelişli, 2019) is mainly defined as teachers’ perception of the opportunity and right to make choice independently in teaching and scientific research, in other words, it means whether the college English teachers can perceive such freedom of decision making in terms of teaching content and teaching methods, the style of managing the classroom, the ways to evaluate students’ performance (0.85), the subjects the teachers are interested in for their academic research (0.82), and training programs according to their own demands (0.62). According to Ebersold et al. (2019), autonomy would have a positive effect on the teachers’ well-being, which is also evidenced by the interviewed teachers’ descriptions in our study.

Impact is connoted as teachers’ perception of their influences on the formulation or implementation of college English education policies (0.70), classroom teaching management and evaluation of students’ performance (0.69). It reflects teachers’ experiences of their agency in solving the problems in their work (0.89), which is quite similar to the results obtained by Spreitzer (1995) and Zimmerman (1995) and is the essential dimension of psychological empowerment. If teachers feel they are not important in the colleagues and the leaders’ eyes, they would have a lower level of psychological empowerment, which may negatively affect their initiative and enthusiasm in participating in decision-making and school management. The college English teachers interviewed in our study admitted they frequently feel their lowering impact and show their indifference to school affairs due to the marginalization of English teaching in college though they are actually eager to speak loudly for such rights.

Status is described as the teachers’ perception of colleagues’ attitudes, evaluation and viewpoints toward themselves in the work. Within the teacher’s community of practice, the interpersonal relationship is critical to teachers’ judgment of their status, which was mainly embodied as whether the colleagues would speak highly of them (0.65), or hold a positive attitude toward their performance (0.72), or whether the colleagues accept, appreciate, respect or support them (0.70). Though it is a bit different from the findings by Bardach et al. (2021) where psychological characteristics matter much to teacher’s interpersonal relations, the essence is similar because status in the interpersonal relationship establish proportionately to the content of the teacher’s psychological empowerment. Therefore, college English teachers’ positive perception of their status would stimulate and thus be transferred into the psychological power which would help build the positive and upward relationship and attitudes for their professional development.

In this study we conducted an empirical study on the development of CETPE’s scale; and the CETPE is found to be systematically composed of the teachers’ positive perception and experience of work meaningfulness, teaching autonomy, occupational competence, professional impact and social status. The college English teachers-oriented and Chinese context-based scale developed in this study would be applied effectively to evaluate their psychological empowerment.

The CETPE scale is expected to systematically innovate a promising path for teachers’ professional development. At the national level, specific measures such as official interpretation and policy making should be taken to exclaim the significance of college English teaching in higher education, improving teachers’ perception of their work meaningfulness and enhancing their professional identity so as to provide impetus for teachers’ professional development. At the school’s level, a positive school organizational atmosphere should be built to improve the overall and various dimensions of teachers’ psychological empowerment. Bearing in mind the purpose of serving teachers’ professional development, the school would improve for the teachers the interrelationship such as the leaders’ support, colleague communication and teacher-student interaction, and better the mechanism and regulations such as participation in decision-making, recognition of evaluation, and clarification of goals (Clément et al., 2020). At the personal level, it would be quite essential for the individual teachers to do self-inspection and self-reflection based on their own personalities and the environment they are in Xu and Lei (2020). The college English teachers should be fully aware of the requirements involved in their professional identity, and reflect and explore the capacity they are equipped with to realize the value of their work so as to develop and improve themselves in study and work (Amor et al., 2021).

The limitations are worthy of mentioning for the sake of further research: the samples collected are not large and broad enough to reflect CETPE’s all-sided connotation; and there is a lack of diachronic tracking data which would fully reflect its dynamic characteristics; it was difficult to test the criterion validity of our scale for lack of authoritative existing scales concerning college English teachers. Further research needs to address the deeper and wider contents in new ecology of college English teaching, and address the its change with time and context, namely, the dynamics of CETPE.

## Data Availability Statement

The original contributions presented in the study are included in the article/supplementary material, further inquiries can be directed to the corresponding author/s.

## Ethics Statement

The studies involving human participants were reviewed and approved by the Ethics Committee of Anhui University of Science and Technology. The patients/participants provided their written informed consent to participate in this study. Written informed consent was obtained from the individual(s) for the publication of any potentially identifiable images or data included in this article.

## Author Contributions

PL was responsible for designing and performing the empirical study and writing the article. JX was responsible for theoretical guidance and article quality improving. Both authors contributed to the article and approved the submitted version.

## Conflict of Interest

The authors declare that the research was conducted in the absence of any commercial or financial relationships that could be construed as a potential conflict of interest.

## Publisher’s Note

All claims expressed in this article are solely those of the authors and do not necessarily represent those of their affiliated organizations, or those of the publisher, the editors and the reviewers. Any product that may be evaluated in this article, or claim that may be made by its manufacturer, is not guaranteed or endorsed by the publisher.

## References

[B1] Amor A. M. Xanthopoulou D. Calvo N. Vázquez P. A. (2021). Structural empowerment, psychological empowerment, and work engagement: a cross-country study. *Eur. Manag. J.* 39 779–789. 10.1016/j.emj.2021.01.005

[B2] Baleghizadeh S. Goldouz E. (2016). The relationship between Iranian EFL teachers’ collective efficacy beliefs, teaching experience and perception of teacher empowerment. *Cogent Educ.* 3 1–15. 10.1080/2331186X.2016.1223262

[B3] Bandura A. (1978). Self-efficacy: toward a unifying theory of behavioral change. *Adv. Behav. Res. Ther.* 1 139–161. 10.1016/0146-6402(78)90002-4 847061

[B4] Bardach L. Klassen R. M. Perry N. E. (2021). Teachers’ psychological characteristics: do they matter for teacher effectiveness, teachers’ well-being, retention, and interpersonal relations? an integrative review. *Educ. Psychol. Rev.* 34 259–300. 10.1007/s10648-021-09614-9

[B5] Bin J. N. (2017). Psychological empowerment on organizational commitment as perceived by Saudi academics. *World J. Educ.* 7 83–92. 10.5430/wje.v7n1p83

[B6] Borg S. Liu Y. D. (2013). Chinese college English teachers’ research engagement. *TESOL Q.* 47 270–299. 10.1002/tesq.56

[B7] Byrne B. M. (2001). *Structureal Equation Modeling with Amos: Basic Concepts, Applications and Programming.* Mahwah, NJ: Lawrence Erlbaum Associates.

[B8] Cao T. Lu N. (2007). *Partnership and Teacher Empowerment: A New Perspective for Teacher Professional Development.* Beijing: Educational Science Press.

[B9] Chaoping L. Xiaoxuan L. Kan S. Xuefeng C. (2006). Psychological empowerment: measurement and its effect on employee work attitude in China. *Acta Psychol. Sin.* 38 99–106.

[B10] Clark L. A. Watson D. (1995). Constructing validity: basic issues in objective scale development. *Psychol. Assess.* 7 309–319. 10.1037/1040-3590.7.3.309 PMC6754793 30896212

[B11] Clément L. Fernet C. Morin A. J. S. Austin S. (2020). In whom college teachers trust? On the role of specific trust referents and basic psychological needs in optimal functioning at work. *High. Educ.* 80 511–530. 10.1007/s10734-019-00496-z

[B12] Conger J. A. Kanungo R. N. (1988). The empowerment process: integrating theory and practice. *Acad. Manag. Rev.* 13 471–482. 10.5465/AMR.1988.4306983

[B13] Dee J. R. Henkin A. B. Duemer L. (2003). Structural antecedents and psychological correlates of teacher empowerment. *J. Educ. Adm.* 41 257–277. 10.1108/09578230310474412

[B14] Ding J. (2021). The impact of psychological empowerment on turnover intention in Chinese university counselors: the mediation role of burnout and the moderating role of professional identity. *Curr. Psychol.* 40 1–10. 10.1007/s12144-021-01955-6

[B15] Dörnyei Z. (2007). *Research Methods in Applied Linguistics: Quantitative, Qualitative and Mixed Methodologies.* Oxford: Oxford University Press.

[B16] Ebersold S. Rahm T. Heise E. (2019). Autonomy support and well-being in teachers: differential mediations through basic psychological need satisfaction and frustration. *Soc. Psychol. Educ.* 22 921–942. 10.1007/s11218-019-09499-1

[B17] Feiz D. Dehghani Soltani M. Farsizadeh H. (2019). The effect of knowledge sharing on the psychological empowerment in higher education mediated by organizational memory. *Stud. High. Educ.* 44 3–19. 10.1080/03075079.2017.1328595

[B18] Gkorezis P. Hatzithomas L. Petridou E. (2011). The impact of leader’s humor on employees’ psychological empowerment: the moderating role of tenure. *J. Manag. Issues* 23 83–95.

[B19] Hair J. F. Babin B. J. Anderson R. E. Black W. C. (2018). *Multivariate Data Analysis*, 8th Edn. Boston, MA: Cengage Learning EMEA.

[B20] Huang Y. T. Guo M. (2019). Facing disadvantages: the changing professional identities of college English teachers in a managerial context. *System* 82 1–12.

[B21] Khany R. Tazik K. (2015). On the relationship between psychological empowerment, trust, and Iranian EFL teachers’ job satisfaction: the case of secondary school teachers. *J. Career Assess.* 24 112–129. 10.1177/1069072714565362

[B22] Kiliańska G. P. (2012). Empowering EFL teachers with interpretive skills: analysis of student teachers’ self-reports. *Hum. Lang. Teach.* 14 1–9.

[B23] Krippendorff K. (2014). *Content Analysis: An Introduction to Its Methodology.* Thousand Oaks, CA: Sage Publications, Inc.

[B24] Lee A. N. Nie Y. (2017). Teachers’ perceptions of school leaders’ empowering behaviours and psychological empowerment: evidence from a Singapore sample. *Educ. Manag. Adm. Leadersh.* 45 260–283. 10.1177/1741143215578448

[B25] Lei M. Medwell J. (2020). How do English language teachers understand the idea of professional development in the recent curriculum reforms in China? *Asia Pac. J. Educ.* 40 401–417. 10.1080/02188791.2020.1717440

[B26] Maeroff G. I. (1989). The principle of teacher empowerment. *Educ. Digest* 54 6–9.

[B27] Mishra A. K. Spreitzer G. M. (1998). Explaining how survivors respond to downsizing: the roles of trust, empowerment, justice, and work redesign. *Acad. Manag. Rev.* 23 567–588. 10.5465/amr.1998.926627

[B28] Morris V. G. Nunnery J. A. (1993). *Teacher Empowerment in a Professional Development School Collaborative: Pilot Assessment.* Memphis, TN: Center for Research in Educational Policy, College of Education, Memphis State University.

[B29] Orakcı S. Gelişli Y. (2019). The effect of the application of learning activities based on learner autonomy on the 6th Grade Students’ English achievements, attitudes, and learner autonomy. *Int. J. Curric. Inst.* 11 269–292.

[B30] Revilla M. Toninelli D. Ochoa C. Loewe G. (2016). Do online access panels need to adapt surveys for mobile devices? *Internet Res.* 26 1209–1227. 10.1108/IntR-02-2015-0032

[B31] Sagnak M. (2012). The empowering leadership and teachers’ innovative behavior: the mediating role of innovation climate. *Afr. J. Bus. Manag.* 6 1635–1641. 10.5897/AJBM11.2162

[B32] Seibert S. E. Wang G. Courtright S. H. (2011). Antecedents and consequences of psychological and team empowerment in organizations: a meta-analytic review. *J. Appl. Psychol.* 96 981–1003. 10.1037/a0022676 21443317

[B33] Short P. M. Rinehart J. S. (1992). School participant empowerment scale: assessment of level of empowerment within the school environment. *Educ. Psychol. Meas.* 52 951–960. 10.1177/0013164492052004018

[B34] Spreitzer G. M. (1995). Psychological empowerment in the workplace: dimensions, measurement, and validation. *Acad. Manag. J.* 38 1442–1465. 10.5465/256865 256865

[B35] Thomas K. W. Velthouse B. A. (1990). Cognitive elements of empowerment: an “interpretive” model of intrinsic task motivation. *Acad. Manag. Rev.* 15 666–681. 10.5465/amr.1990.4310926

[B36] Viladrich C. Angulo-Brunet A. Doval E. (2017). A journey around alpha and omega to estimate internal consistency reliability. *Anal. Psicol. Ann. Psychol.* 33 755–782. 10.6018/analesps.33.3.268401

[B37] Wang A. Wei X. Zhang Q. (2021). College english classroom teaching activities in smart classrooms environment —based on a case study of seven college English teachers. *Mod. Educ. Technol.* 31 68–76. 10.3969/j.issn.1009-8097.2021.10.008

[B38] Wang J. L. Zhang D. J. Jackson L. A. (2013). Influence of self-esteem, locus of control, and organizational climate on psychological empowerment in a sample of Chinese teachers. *J. Appl. Soc. Psychol.* 43 1428–1435. 10.1111/jasp.12099

[B39] Wang J. L. Zhang D. J. (2011). Development of a psychological empowerment questionnaire for teachers at elementary and secondary schools in China. *Psychol. Dev. Educ.* 1 105–111. 10.16187/j.cnki.issn1001-4918.2011.01.015

[B40] Wang R. (2015). *Study on Teachers’ Psychological Empowerment in the College Organizational Environment.* Beijing: China Social Sciences Press.

[B41] Wang R. W. Liu J. L. (2014). Organizational environment, psychological empowerment and organizational commitment: based on empirical analysis of university teachers’ individual evaluation. *J. Dalian Univ. Technol. Soc. Sci.* 35 126–132. 10.19525/j.issn1008-407x.2014.03.023

[B42] Xu J. F. (2020). On the research of second language teacher psychology. *Foreign Lang. Res.* 214 56–62.

[B43] Xu J. F. Lei P. F. (2020). A narrative study on teaching-researching integration in college English teachers’ professional development. *Foreign Lang. China* 17 62–68. 10.13564/j.cnki.issn.1672-9382.2020.06.010

[B44] Yao J. You Y. Zhu J. (2019). Principal–teacher management communication and teachers’ job performance: the mediating role of psychological empowerment and affective commitment. *Asia Pac. Educ. Res.* 29 365–375. 10.1007/s40299-019-00490-0

[B45] Zhu J. Yao J. Zhang L. (2019). Linking empowering leadership to innovative behavior in professional learning communities: the role of psychological empowerment and team psychological safety. *Asia Pac. Educ. Rev.* 20 657–671. 10.1007/s12564-019-09584-2

[B46] Zimmerman M. A. (1995). Psychological empowerment: issues and illustrations. *Am. J. Commun. Psychol.* 23 581–599. 10.1007/BF02506983 8851341

